# Phytochemical Characterization and Biological Evaluation of *Origanum vulgare* L. Essential Oil Formulated as Polymeric Micelles Drug Delivery Systems

**DOI:** 10.3390/pharmaceutics14112413

**Published:** 2022-11-08

**Authors:** Larisa Bora, Tobias Burkard, Martina Herrero San Juan, Heinfried H. Radeke, Ana Maria Muț, Lavinia Lia Vlaia, Ioana Zinuca Magyari-Pavel, Zorița Diaconeasa, Sonia Socaci, Florin Borcan, Brigitta Kis, Delia Muntean, Cristina Adriana Dehelean, Corina Danciu

**Affiliations:** 1 Department of Pharmacognosy, Victor Babes University of Medicine and Pharmacy, Eftimie Murgu Square, No. 2, 300041 Timisoara, Romania; 2 Research Center for Pharmaco-Toxicological Evaluation, Victor Babes University of Medicine and Pharmacy, Eftimie Murgu Square, No. 2, 300041 Timisoara, Romania; 3 Pharmazentrum Frankfurt/ZAFES, Institute of General Pharmacology and Toxicology, Hospital of the Goethe University, 60596 Frankfurt am Main, Germany; 4 Department II—Pharmaceutical Technology, Formulation and Technology of Drugs Research Center, Victor Babes University of Medicine and Pharmacy, Eftimie Murgu Square, No. 2, 300041 Timisoara, Romania; 5 Department of Food Science and Technology, Faculty of Food Science and Technology, University of Agricultural Science and Veterinary Medicine, Calea Manastur, 3-5, 400372 Cluj-Napoca, Romania; 6 Department of Analytical Chemistry, Victor Babes University of Medicine and Pharmacy, Eftimie Murgu Square, No. 2, 300041 Timisoara, Romania; 7 Centre for Gene and Cellular Therapies in the Treatment of Cancer-OncoGen, Clinical County Hospital of Timisoara, Liviu Rebreanu Blvd. 156, 300736 Timisoara, Romania; 8 Department of Microbiology, Victor Babes University of Medicine and Pharmacy, Eftimie Murgu Square, No. 2, 300041 Timisoara, Romania; 9 Multidisciplinary Research Center on Antimicrobial Resistance, Victor Babes University of Medicine and Pharmacy, Eftimie Murgu Square, No. 2, 300041 Timisoara, Romania; 10 Department of Toxicology and Drug Industry, Victor Babes University of Medicine and Pharmacy, Eftimie Murgu Square, No. 2, 300041 Timisoara, Romania

**Keywords:** *Origanum vulgare* L. essential oil, GC-MS, antioxidant, polymeric micelles, keratinocytes, dendritic cells

## Abstract

This study presents phytochemical characterization and biological evaluation of *Origanum vulgare* L. essential oil (OEO) formulated as polymeric micelles drug delivery systems as a possible non-invasive approach for the management of skin tags. GC-MS analysis of Romanian OEO revealed the identification and quantification of 43 volatile compounds (thymol and carvacrol being the main ones). The antioxidant activity was shown by four consecrated methods: CUPRAC, ABTS, ORAC and DPPH. OEO was incorporated by micellar solubilization into a binary hydrogel based on a Pluronic F 127/L 31 block-copolymers mixture. The pH, consistency, spreadability, particle size, polydispersity index and zeta potential of the OEO-loaded poloxamer-based binary hydrogel (OEO-PbH) were investigated. OEO-PbH was skin compatible in terms of pH and exhibited adequate spreadability and consistency. The minimal inhibitory concentrations of the tested OEO were similar to those obtained for the formulation, lower (2.5 µg/mL) for yeast and higher (40–80 µg/mL) for Gram-negative bacilli. As keratinocytes are among main components of skin tags, an in vitro evaluation was conducted in order to see the effect of the formulation against HaCaT human keratinocytes. OEO-PbH decreased HaCaT cells migration and proliferation and elicited a cytotoxic and pro-apoptotic effect in a dose- and time-dependent manner. No harmful effect on the viability of dendritic cells (DCs) was detected following the incubation with different concentrations (0–200 µg/mL) of the 5% formulation. Treatment in inflammatory DCs (+LPS) indicated a decrease in cytokine production of IL-6, TNF-α and IL-23 but no significant effect on IL-10 in any of the tested concentrations.

## 1. Introduction

From ancient times, people have tried to treat and solve health issues using natural resources at hand. The first writings were found in China around 3000 B.C. and were wrote by Emperor Cho Chin Ken. Other references were found in a papyrus from Egypt which contains over 1700 medicinal plants descriptions. Therefore, the World Health Organization recognizes phytotherapy as an important element in the health field and supports the scientific approach of plant-based therapy [1]. Because of the fact that the use of plant based products has demonstrated over the years efficacy and safety when used as prescribed, currently scientists are working to improve phytotherapy practice in order to offer new healthcare complementary and/or alternative treatments [2]. Thus, despite the fulminant development of the pharmaceutical industry, phytotherapy remains a resourceful therapeutic option [3]. In this field, researchers’ attention has been focused on the use of herbal medicinal products with pharmaceutical properties, both as different types of extracts or as pure isolated phytocompounds as well as essential oils. As is well known, volatile oils are complex blends of compounds with low molecular weight, having numerous pharmacological activities, including antibacterial, antiviral, antifungal, antiprotozoal, anti-inflammatory, antimutagenic, anticancer and antidiabetic properties [4].

One of the most appreciated and valued aromatic plants, rich in volatile oils and well known for its pharmacological activities, is represented by *Origanum vulgare* L., a *Lamiaceae* species native to the European, African and also Asian continents. It covers several subspecies that can be divided into species rich in essential oils (subsp. *glandulosum*, subsp. *gracile*, subsp. *hirtum*) and species poor in essential oils (subsp. *virens*, subsp. *viridulum*, subsp. *vulgare*) [5]. The origin of the name *Origanum* comes from two Greek words: *oros*, which means mountains, and *ganos*, which means joy, an allusion to the region where the plant is cultivated [6]. The most prevailing *Origanum vulgare* L. species in Romania is *O. vulgare* L. subsp. *vulgare* [7]. The volatile oil composition fluctuates according to geographical area, surroundings and growth conditions, stage of plant maturity, altitude and harvesting time [8]. Therefore, the flowering phase (middle of June to late August) has been proven to be the most proper period to obtain huge quantities of *O. vulgare* L. volatile oil [9].

The chemical composition of the ethanolic extract of *Origanum vulgare* L. includes mainly phenolic acids (rosmarinic, chlorogenic, caffeic acids) and flavonoids (luteolin, quercetin, hyperoside) [7,10]. The most well expressed volatile oil compounds are monoterpens and sesquiterpene hydrocarbons, especially thymol, carvacrol, γ-terpinene, linalool, *p*-cymene [5,11]. It is important to mention that both the climatic factors as well as the soil conditions can lead to different chemotype composition. Therefore, plants originating from the Continental climate are characterised mainly by an active sabinyl-pathway, while plants cultivated in regions with Mediterranean climate present an efficient cymyl and/or linalool pathway [12].

Traditionally, oregano is used worldwide as a spice in gastronomy and as part of the flavour in soft drinks or bitter tinctures. Due to its intensive fragrance, *Origanum* species are also used in the cosmetic industry and dermatology [6]. In traditional medicine, oregano is used as tinctures, infusions and ointments in order to treat digestive, respiratory and dermatological disorders [5]. It can also be used as a menstruation regulator and in reproductive problems [6].

Besides the huge number of articles concerning *Origanum vulgare* L.’s antimicrobial properties, many in vitro and in vivo studies focus on other biological activities, including antiproliferative, antioxidant, antihyperglycemic, vasoprotective and anti-inflammatory effects, both for preventive and therapeutic purposes [6,13].

It is acknowledged that OEO has a strong antibacterial, antifungal and antiparasitic effect due to the phenolic compounds thymol and carvacrol, which interfere with the pathogen cell membrane and cause functional and structural damage. The essential oil acts on bacteria such as *Pseudomonas aeruginosa* (*P. aeruginosa*), *Staphylococcus aureus* (*S. aureus*), *Streptococcus* sp., *Propionibacterium acnes* (*P. acnes*), *Escherichia coli* (*E. coli*), *Helicobacter pylori* (*H. pylori*) and *Klebsiella* sp. [5,14,15], and it has a strong antifungal effect on *Candida albicans* (*C. albicans*) [16] and can also be used on parasites such as *Cryptosporidium parvum* (*C. parvum*), *Echinococcus granulosus* (*E. granulosus*) and *Trypanosoma cruzi* (*T. cruzi*) [17].

The antiproliferative activity of OEO was intensively studied on several types of carcinoma, including liver and renal tumors, and the conclusions are promising. Carvacrol and thymol seem to have a selective anticancer potential against tumor cells, without influencing the healthy cells [18,19,20,21].

OEO has been reported to possess major anti-inflammatory activity due to the presence of carvacrol, with a mechanism that involves the inhibiton of pro-inflammatory cytokines (interleukin-1β (IL-1β), interleukin-6 (IL-6), interleukin-8 (IL-8), tumoral necrosis factor (TNF-α)), NADPH oxidase, lypoxigenase, and reactive oxigen species. Thus, it can be a candidate to give an alternative therapeutic approach for chronic diseases led by inflammation, such as Alzheimer, Parkinson, cancer, skin disorders, diabetes, and other metabolic syndromes [5,6,22,23]. At the same time, regarding the immunomodulatory effect, thymol and carvacrol substantiated a potent inhibition on both whole blood phagocytes and isolated polymorphonuclear leukocytes (PMNs), demonstrating a solid inhibition on oxidative burst comparable to that of synthetic drugs such as ibuprofen [24,25].

Poloxamers, also known as Pluronics^®^, are biocompatible nonionic hydrosoluble triblock copolymers consisting of two polar blocks of polyoxyethylene and a central non-polar block of polyoxypropylene, which are responsible for the amphiphilic and tensioactive properties of these polymers [26]. Moreover, poloxamers are considered smart polymers for their thermoresponsive property, exhibiting sol-gel transition in aqueous solutions with increasing temperature and consequently allowing them to obtain thermosensitive hydrogels [27]. Due to their amphiphilic features, poloxamers form spontaneously in aqueous solution ordered aggregates, usually micelles, possessing the ability to solubilize hydrophobic compounds, such as those contained in essential oils. Furthermore, modern drug delivery systems enhance the bioavailability and therapeutic efficacy of volatile oils [28,29]. Considering the abovementioned features and their non-toxic and non-irritant character (they are FDA approved and listed in the European and US Pharmacopoeias), the poloxamers are a suitable option as solubilizers and gelling agents in the formulation of topical semisolid drug carriers for poorly hydrosoluble bioactive compounds [30,31].

This study aimed to design a modern pharmaceutical formulation-type poloxamer-based binary hydrogel having as active ingredient OEO and to evaluate in vitro some biological activities (antiproliferative/pro-apoptotic effect against HaCaT human keratinocytes, antimicrobial activity against selected strains, immunomodulatory potential) as a starting study for further research of this formulation as a possible non-invasive approach used for the elimination of skin tags.

## 2. Materials and Methods

### 2.1. Plant Materials

The volatile oil obtained from the species *Origanum vulgare* var. *vulgare*, collected from the western part of Romania and identified in the Department of Pharmacognosy, University of Medicine and Pharmacy “Victor Babes” Timisoara (specimen number OV 5/2020) was extracted by hydro distillation. Briefly, 100 g of shredded dry vegetable product was weighed and introduced into the extraction flask. The refrigerant and the collecting cup were attached, the continuous flow of cold water was released, and the heating nest was turned on. When the mixture reached the boiling point (100 °C), hydro distillation began. The extraction time was approximately 1 h. After the extraction was ready, the aromatic water, together with the volatile oil, was decanted into a separating funnel. It was left at rest 2–3 h and then the two phases were separated.

### 2.2. Gas Chromatography-Mass Spectroscopy (GC-MS) Quantitative Analysis of the Volatile Compounds

The volatile profile of the essential oil samples was performed by GC-MS using a GC-MS (QP-2010 model, Shimadzu Scientific Instruments, Kyoto, Japan) equipped with a Combi-PAL AOC-5000 autosampler (CTC Analytics, Zwingen, Switzerland) and a capillary column (ZB 5 ms, 30 m × 0.25 mm i.d. × 0.25 µm thickness, Phenomenex, Torrance, CA, USA). An aliquot from the essential oil sample was diluted in hexane and 1 μL was injected in the GC-MS at a split ratio of 1:50. The separation of the volatile constituents was performed using the following column temperature program: the initial temperature of 50 °C (maintained for 2 min) was increased to 160 °C at a rate of 4 °C/min and then to 250 °C with 15 °C/min and hold for 10 min. The carrier gas was helium at a constant flow of 1 mL/min. The temperature for injector, ionic source, and interface was set at 250 °C. The detection was performed on a quadrupole mass spectrometer operating in full scan (40–500 *m*/*z*), with electron impact (EI) as ion source at an ionization energy of 70 eV. The tentative identification of the volatile compounds was achieved by comparing their recorded mass spectra and the fragmentation patterns with those from the software’s NIST27 and NIST147 mass spectra libraries (considering a minimum similarity of 85%). The relative percentage of each compound was estimated as a fraction of its integrated ion area from the total ion chromatograms (TIC) area (100%).

### 2.3. Determination of the Antioxidant Activity

#### 2.3.1. 2,2-Diphenyl-1-picrylhydrazyl (DPPH) Radical Scavenging Assay

The DPPH scavenging activity assay was performed according to a protocol published previously [32]. Briefly, a DPPH solution (60 μM) was freshly prepared in 95% methanol. The analysis was performed as follows: 2 mL of DPPH solution was allowed to react with 50 μL sample and the absorbance was recorded at 517 nm, for 60 min using a JASCO V-630 spectrophotometer (International Co, Ltd., Tokyo, Japan). The antioxidant activity was calculated as follows:

%DPPH·_scavenging activity_ = (1 − [A_sample_/A_control t = 0_]) × 100. (1)

#### 2.3.2. 2,2-Azino-bis-3-ethylbenzothiazoline-6-sulfonic Acid (ABTS) Radical Scavenging Assay

The ABTS assay was done by an adapted protocol to 96-well microplate [33,34]. The ABTS^+^ solution was produced by reacting 7 mM ABTS stock solution with 2.45 mM potassium persulfate (final concentration) for 12–16 h in the dark at room temperature. The working solution was prepared by diluting the ABTS stock solution with EtOH to an absorbance of 0.70 ± 0.02 at 734 nm. The samples and Trolox standards (20 μL) were mixed with the ABTS working solution in 96-well microplate. After 6 min of incubation at 30 °C, the absorbance (734 nm) was read with a microplate reader (BioTek Instruments, Winooski, VT, USA). The results were expressed as µmol Trolox equivalents per g (TE μmol/g).

#### 2.3.3. Cupric Ion Reducing Antioxidant Capacity (CUPRAC) Assay

Before measurements, a 10 mM solution of copper (II) in water (34.1 mg CuCl_2_·2H_2_O in 20 mL water) and a 7.5 mM solution of neocuproine in ethanol (31.2 mg neocuproine in 20 mL ethanol) were prepared. A buffer solution of 1.0 M ammonium acetate, pH 7.0, was also prepared (19.27 g NH_4_Ac in 250 mL water), while a 2% solution of methyl-β-cyclodextrin (M-β-CD) was prepared in ethanol. The samples were initially dissolved in M-β-CD (2% (*v*/*v*) solutions) and then subjected to two serial dilutions with the same solvent to obtain concentrations in the 0.01–2% range. The assay was conducted by mixing 50 µL of copper (II) solution, 50 µL of neocuproine solution, 70 µL of buffer solution of ammonium acetate, and 30 µL of diluted sample or standard in a 96-well microplate. The microplate was incubated at 25 °C for 60 min and the absorbance of samples/standards was recorded at 450 nm against the blank. The calibration curve obtained from the standards was used to calculate the total antioxidant capacity (TAC) of the serial dilutions. The results were expressed as µmol of Trolox equivalents per mL oil (µmol TE/mL) [35,36].

#### 2.3.4. Oxygen Radical Absorption Capacity (ORAC) Assay

The ORAC assay was conducted using a 96-well microplate reader. Trolox standard solution was prepared (6.25, 1.25, 25, 50 µmol/L) with a phosphate buffer (pH 7.4). Fluorescein (0.12 µmol/L) was used as substrate and 2,2′-azobis(2-amidinopropane) dihydrochloride (AAPH) (40 mmol/L) was used as radical generator. Trolox standard solution and the oil (20 µL) were added in each of the 96-well black microplates, and afterwards 120 µL fluorescein was added at phosphate buffer solution. The microplate was incubated at 37 °C for 20 min and then 60 µL AAPH were added in each well. A standard curve was drawn and ORAC value of the tested compounds was obtained as TE. ORAC values were reported as Trolox equivalents expressed as µmol TE/mL. The intensity of fluorescence was monitored (excitation: 485 nm, emission: 525 nm) [37].

### 2.4. Preparation and Characterization of the Polymeric Micelles Drug Delivery System

#### 2.4.1. Materials and Preparation of the Poloxamer-Based Binary Hydrogels

Poloxamer 407 (Pluronic F127) and Pluronic L 31 were purchased from Sigma-Aldrich-Chemie (Steinheim, Germany). Poloxamer-based binary hydrogels were prepared by the cold method [38]. To prepare blank poloxamer-based hydrogel (B-PbH), accurately weighed amounts of P127 (20% *w*/*w*) and P31 (1% *w*/*w*) were added to purified water cooled to 4 °C. The mixture was kept in the refrigerator at least 24 h and periodically stirred until a clear, homogenous solution was obtained, thus ensuring the complete dissolution of the poloxamers. Poloxamer-based binary formulation containing OEO (5% *w*/*w*) was prepared by the same method, adding the volatile oil to the cold aqueous solution of the two poloxamers under continuous stirring until a clear, homogenous hydrogel was obtained.

#### 2.4.2. Physicochemical Characterization of OEO-PbH and B-PbH

Before performing in vitro and in vivo evaluations, the two poloxamer-based binary hydrogels were subjected to macroscopic examination, pH determination, and rheological measurements.

The compendial potentiometric method [39] was used to determine the pH of poloxamer-based binary hydrogels. Measurements were carried out using a pH-meter (Sension™ 1 portable digital pH meter, Hach Company, Columbus, OH, USA) at a temperature of 25 ± 2 °C. The weighed amount of hydrogel (1 g) was dispersed in purified water (20 mL) under stirring for 15 min at room temperature. The pH of the obtained clear solution was measured three times for each sample.

For rheological characterization of the hydrogels, the compendial penetrometric method and the parallel-plate method were selected to measure the penetration degree and the spreadability, respectively, as consistency related parameters. Penetrometric and spreadability experiments were performed with a penetrometer (PNR 12, Petrolab, Speyer, Germany) equipped with a microcone and suitable container and the del Pozo Ojeda-Suñé Arbussá extensometer, following the procedures described in the literature [40,41]. All rheological experiments were carried out in triplicate at 25 ± 2 °C.

The sizes of the particles as well as their surface charge were measured using diluted samples and a Cordouan Zetasizer module (Cordouan Technol., Pessac, France) containing a Vasco Analyzer for particles size and a Wallis Analyzer for Zeta potentials. There were set the following parameters for size measurements: temperature (25 °C), time interval (20 ± 10% µs), number of channels (400 ± 10%), DTC position (up), laser power (between 50 ± 10%), acquisition (continuous mode), and analysis mode (cumulants). The following parameters were chosen for zeta potentials determination: cuvette type (polystyrene cuvette wavelength range 340–750 nm), temperature (25 °C), laser power (60%), resolution (medium, 0.8 Hz), applied field (automatic), and Henry function (Smoluchowski). The measurements were performed in duplicate.

### 2.5. Antimicrobial Activity Evaluation

#### 2.5.1. Bacterial Strains

Seven bacterial and fungus strains from American Type Culture Collection (Manassas, Virginia, USA) were tested for their susceptibility to OEO-PbH and OEO. The tested strains were represented by *Streptococcus pyogenes* (*S. pyogenes*) ATCC 19615, *S. aureus* ATCC 25923, *Enterococcus faecalis* (*E. faecalis*) ATCC 51299, *E. coli* ATCC 25922, *P. aeruginosa* ATCC 27853, *C. albicans* ATCC 10231, and *Candida parapsilosis* (*C. parapsilosis*) ATCC 22019.

#### 2.5.2. Determination of the Minimal Inhibitory Concentration (MIC), Minimal Bactericidal Concentration (MBC), and Minimal Fungal Concentration (MFC)

The antimicrobial properties of OEO-PbH was evaluated by dilution method according to the European Committee on Antimicrobial Susceptibility Testing (EUCAST), Clinical Laboratory and Standard Institute (CLSI), and other previous studies [42,43,44,45,46].

Seven microorganisms were tested for their susceptibility to OEO-PbH. From each microbial strain, standardized suspensions were prepared to a concentration of 0.5 MacFarland, approximatively 10^8^ CFU/mL (CFU—colony forming unit). Then, broth was added (Mueller–Hinton or Mueller–Hinton supplemented with defibrinated horse blood and β-NAD, bioMérieux, Craponne, France) and serial dilutions were obtained from OEO-PbH, obtaining a final microbial density of approximately 10^5^ CFU/mL. After incubating at 35–37 °C for 24 h, MIC was read. MIC is defined as the lowest concentration of the test compounds without visible growth. To determine MBC or MFC, a volume of 1 µL from the test tubes with no visible growth was inoculated on solid culture media (Columbia agar + 5% sheep blood or Sabouraud Dextrose Agar, bioMérieux, France). The lowest concentration which killed 99.9% of the microorganisms was established after being incubated at 35–37 °C for 24 h. In the same mode OEO was also tested as positive control and the excipient as negative control.

### 2.6. In Vitro Evaluation of OEO Formulated as Polymeric Micelles Drug Delivery Systems

#### 2.6.1. Cell Culture

The human keratinocytes HaCaT cell line was provided by the University of Debrecen, Hungary. Dulbecco’s Modified Eagle’s Medium (DMEM) high in glucose, supplemented with a mixture of two antibiotics (penicillin/streptomycin 10.000 IU/mL) in order to avoid contamination and fetal bovine serum 10% (FCS) were used to cultivate HaCaT cells. The reagents were purchased from Sigma-Aldrich, Taufkirchen, Germany. The cells were incubated in standard conditions (37 °C, 5% CO_2_).

#### 2.6.2. Cell Viability Assessment by MTT Assay

The antiproliferative activity of OEO-PbH was evaluated against human keratinocytes cell line by means of MTT (3-(4,5-dimethylthiazol-2-yl)-2,5-diphenyltetrazolium bromide) assay. The method was conducted according to the protocol described by Kis et al. [47]. HaCaT cells were plated in 96 multi-wells plates and incubated at 37 °C in 5% CO_2_. After 24 h, the adhered cells were stimulated with OEO-PbH and B-PbH and incubated for 24, 48, and 72 h. After the stimulation process and the incubation time, the cells were treated with 10 µL MTT solution (5 mg/mL). After 3 h of incubation, the formed formazan crystals were solubilized with 100 µL lysis solution. The MTT kit was provided from Sigma-Aldrich. After 30 min, the absorbance was read at 570 nm by a microplate spectrophotometer reader (BioRad, xMarkTM Microplate, Serial No. 10578, Tokyo, Japan).

#### 2.6.3. Cell Migration Assessment by Scratch Assay

The migration capacity of HaCaT cells after stimulation with OEO-PbH was evaluated by scratch assay. The method was performed as previously described [47]. HaCaT cells were seeded at a density of 2 × 10^5^ cells/well into 12-well plates and cultured until a percent of 90% confluence was obtained. Afterwards, a sterile pipette tip was used to detach the adhered cells. The wells were washed with Phosphate Buffer Saline (PBS) in order to remove the scratched cells and debris. Subsequently, the cells were stimulated with OEO-PbH and B-PbH and several images were taken with Olympus IX73 inverted microscope with DP74 camera (Olympus, Tokyo, Japan) at 0 h and 24 h. The cell growth was analyzed with cell Sense Dimension software and the scratch closure rate was calculated with the following formula:

(2) $$\mathrm{Scratch}\;\mathrm{closure}=\left[\frac{A_{t0}-{\;A}_{t}}{A_{t0}}\right]\;\times \;100\;\mathrm{rate}$$

where A_t0_ represents the scratch area at time 0 and A_t_ represents the scratch area at 24 h.

#### 2.6.4. Cell Cytotoxicity Assessment by LDH Assay

The cytotoxic effect of OEO-PbH was evaluated against HaCaT cells by means of LDH (lactate dehydrogenase) assay (CyQUANT, Thermo Fisher Scientific, Boston, MA, USA) as previously described by Minda et al. [48]. The cells were plated at a density of 5 × 10^3^ cells/well in a 96-well microplate. After 24 h, the adhered cells were stimulated with OEO-PbH and B-PbH and incubated for 72 h. The following steps involved the transfer of a volume of 50 µL from each well into a new 96-well plate and the addition of an equal volume of the reaction mixture in each well of the new plate. After an incubation period of 30 min at room temperature, 50 µL of stop solution were added in each well. xMark Microplate spectrophotometer (BioRad, xMarkTM Microplate, Serial No. 10578, Tokyo, Japan) was used in order to determine the level of LDH release at 490 nm and 680 nm.

#### 2.6.5. Hoechst Staining

This method was conducted in order to evaluate if the OEO-PbH presents a pro-apoptotic effect and was performed as previously described [49]. HaCaT cells were seeded at a density of 1 × 10^5^ cells/well into 12-well plates. After 24 h, the cells were stimulated with OEO-PbH and incubated for 72 h. Further, the samples were removed from the wells and the staining solution was added in each well (dilution 1:2000 in PBS, 100 µL/well). The plate was incubated at room temperature for 10 min, protected from light. Then, the cells went through three washes with PBS. The images were captured with a fluorescence inverted microscope Olympus IX73 with DP74 camera (Olympus, Tokyo, Japan) at a magnification of 20×. Staurosporine (5 µM) was used as an indicator for apoptosis induction and Triton X-100 (0.5%) for necrosis.

### 2.7. Immunomodulatory Effects of OEO-PbH on Human DCs Culture

#### 2.7.1. In Vitro Culture of Human DCs

Buffy coats were obtained from the blood donation center DRK-Blutspendedienst Baden-Württemberg-Hessen, Institut für Transfusionsmedizin und Immunhämatologie Frankfurt am Main, Frankfurt, Germany and were used for the isolation of human peripheral blood mononuclear cells (PBMCs) by density gradient centrifugation using Ficoll–Histopaque 1.077 g/mL density (Sigma-Aldrich, Steinheim, Germany). Isolated PBMCs were seeded at a density of 7.5 × 10^6^ cells/mL into 6-well plates (Greiner bio-one, Frickenhausen, Germany) and cultivated for 2 h in RPMI 1640 + Glutamax supplemented with 50 mM β-mercaptoethanol, 1 mM sodium pyruvate, 100 μg/mL streptomycin, 100 IU/mL penicillin (all from Gibco, Waltham, MA, USA), and 2 nM HEPES (Sigma-Aldrich, Steinheim, Germany) in the presence of 10% autologous and heat inactivated serum. After 2 h of incubation, all non-adherent cells were aspirated, and the remaining adherent monocytes were washed twice with PBS. For the differentiation into DCs, IL-4 (50 U/mL) and granulocyte-macrophage colony stimulating factor (GM-CSF) (80 U/mL) were added to the cell culture for 7 days. To test the effects of the OEO-PbH formulation on DCs, different concentrations (0–200 µg/mL) of OEO-PbH were added in the presence or absence of the inflammatory stimulus lipopolysaccharide (LPS) (250 ng/mL) for additional 24 h. A time schedule for the experimental set-up is provided within the supplementary information (Supplementary Figure S1).

#### 2.7.2. Cell Viability and Apoptosis Assay

The potential cytotoxic effects of OEO-PbH on human DCs were assessed by flow cytometry analysis using DAPI staining for cell viability and Annexin V/7′AAD for early and late apoptosis detection. 1 µM staurosporine (LC Laboratories, Woburn, MA, USA) was used as a positive control for apoptosis stainings. After 24 h of either OEO-PbH +/− 250 ng/mL LPS stimulation or staurosporine treatment, cells were harvested from cell culture plates and were immediately washed twice with Annexin binding buffer (0.1 M NaCl, 25 mM CaCl_2_, 0.1 M HEPES). Subsequently, cells were incubated with 5 µL Annexin V FITC (Immunotools, Friesoythe, Germany) for 15 min at room temperature and protected from light. The cells were pelleted and suspended in 400 μL Annexin-binding buffer containing 100 ng/mL DAPI (Sigma, Merck, Darmstadt, Germany) for cell viability staining and 5 μL of 7′AAD (Invitrogen, Waltham, MA, USA). Samples were acquired within the following 2 h using a FACS Canto II (BD Biosciences, Heidelberg, Germany). A general gating strategy is provided in the supplementary information (Supplementary Figure S2A). Fluorescence minus one (FMO) controls were used to allow proper compensation and gating (Supplementary Figure S2B). For analysis of absolute cell counts, a part of the harvested was analyzed using MACSQuant 10 cell analyzer (Miltenyi Biotech, Bergisch Gladbach, Germany). The flow cytometer performance was regularly controlled using Cytometer Setup and Tracking beads (BD Biosciences, Heidelberg, Germany). Data was analyzed using FlowJo software V10.7.1. For additional assessment of overall viability of cultured DCs, bright-field microscopy images were obtained from a 20× objective of a light microscope (Keyence, Neu-Isenburg, Germany).

#### 2.7.3. Cytokine Measurements

Cell culture supernatants were collected and analyzed by enzyme-linked immunosorbent assay (ELISA) for IL-6, IL-10, TNF-α (all Bio-Techne GmbH, Wiesbaden, Germany), and IL-23 according to the manufacturer’s recommendations.

### 2.8. Statistical Analysis

Statistical analysis was performed by using GraphPad Prism 8.0.1 software (San Diego, CA, USA) in order to analyze the in vitro experiments. One-way ANOVA was used to compare the groups and was followed by Dunnett’s multiple comparison test (* *p* < 0.05; ** *p* < 0.01; *** *p* < 0.001; **** *p* < 0.0001). Data were represented as mean ± standard deviation (SD).

## 3. Results

### 3.1. GC-MS Characterization of OEO

**Figure 1:**
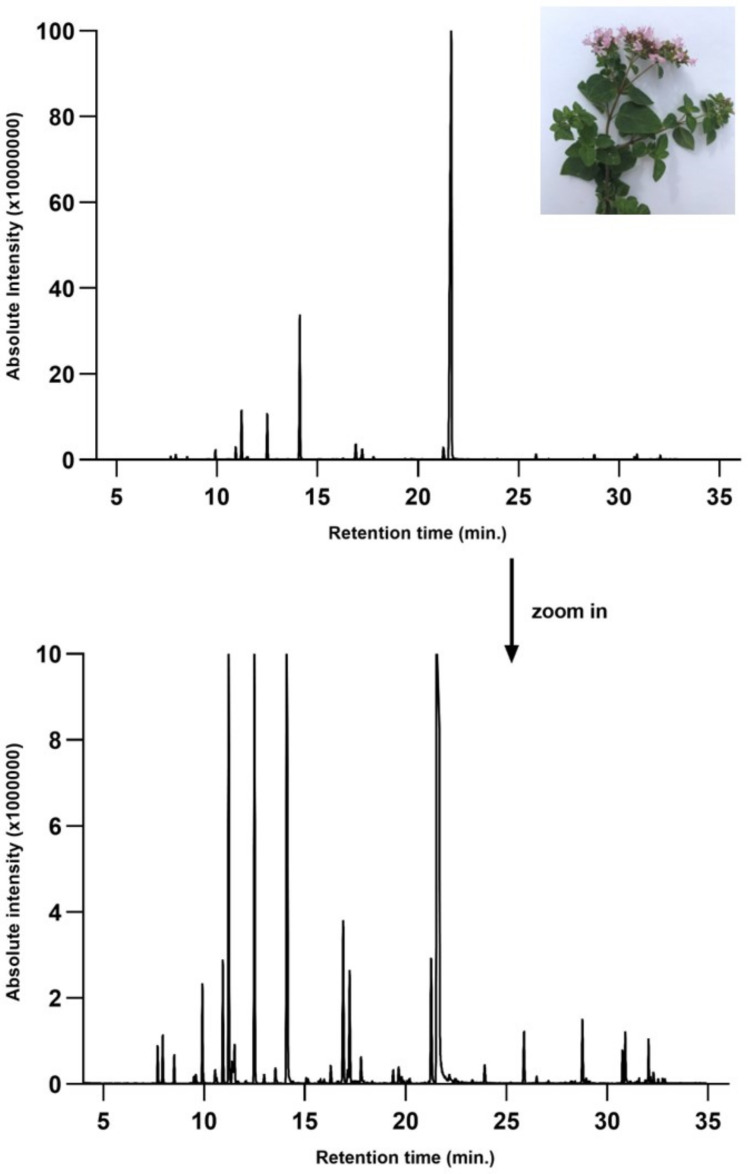
Chromatographic profile of OEO.

The identification of volatile compounds present in OEO was determined by comparing the retention times with those collected in data libraries. The chromatographic profile of OEO is shown in Figure 1.

GC-MS analysis of Romanian OEO revealed the identification and quantification of forty-three volatile compounds, according to their *R*_t_ (Table 1). The most important volatile compounds identified were carvacrol, thymol, β-linalool, *p*-cymene, γ-terpinene, β-bisabolene, caryophyllene, α-terpinene, and β-myrcene, expressed as % of total peak areas.

### 3.2. The Antioxidant Activity of OEO

The antioxidant activity was determined by using four different consecrated methods: CUPRAC, ABTS, ORAC, and DPPH. The first three methods measure the ability of compounds to eliminate different types of radicals by hydrogen transfer (ORAC) and single-electron transfer mechanisms (hydroxyl radicals by CUPRAC) or by a combination (cationic radicals, ABTS method). In order to facilitate a direct comparison of the collected data, the total measured antioxidant capacity for the analyzed oils was compared with that of Trolox standards and the final results were expressed as TE μmol/mL. The values obtained using the four methods are summarized in Table 2.

### 3.3. Physicochemical Characterization of OEO-PbH and B-PbH

#### 3.3.1. Macroscopic Examination

**Figure 2:**
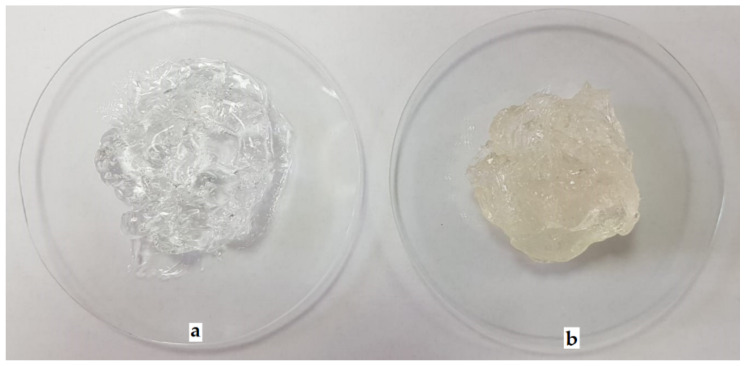
Macroscopic appearance of the experimental poloxamer binary hydrogels: (**a**) B-PbH and (**b**) OEO-PbH.

Visual examination showed that prior to OEO incorporation, B-PbH was clear, colorless, odorless, and transparent (Figure 2a). After OEO incorporation, the hydrogel became yellowish, with a specific odor but maintaining the clear and transparent aspect (Figure 2b), confirming the complete solubilization of the essential oil by the poloxamers mixture.

#### 3.3.2. Determination of pH

OEO-PbH and B-PbH were found to have very slightly acidic pH (6.34 ± 0.05 and 6.47 ± 0.06, respectively).

#### 3.3.3. Rheological Characterization

The mean penetration values of the tested poloxamer binary hydrogels through penetrometry were: 118.0 ± 2.65 mm (B-PbH) and 99.3 ± 1.15 mm (OEO-PbH). It can be observed that the penetration value of the formulation containing essential oil was slightly lower than that of blank formulation, indicating its higher consistency.

**Figure 3:**
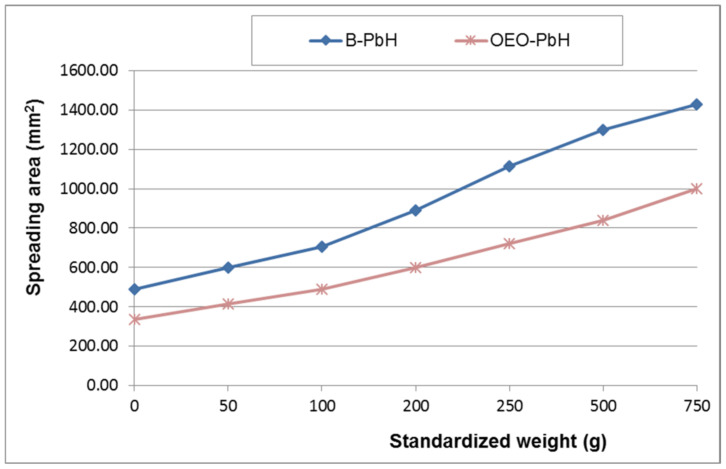
Extensiometric profiles of experimental poloxamer binary hydrogels (B-PbH and OEO-PbH).

Figure 3 shows the results of the spreadability test as extensiometric profiles.

#### 3.3.4. Particle Size and Zeta Potential Determination

The recorded values for the particles size revealed two different populations for B-PbH and OEO-PbH samples with increased polydispersity indexes (PDI), which are specific to heterogeneous systems, while the pure essential oil contains a large population around 635 nm. On the other hand, there is an important difference between the values of zeta potentials, as can be seen in Table 3.

### 3.4. The Antimicrobial Activity of OEO and OEO-PbH

Testing the antibacterial activity of both OEO and OEO-PbH on microbial strains demonstrated their microbicidal effect. Out of the seven tested strains, three were represented by Gram-positive cocci (*S. aureus*, *S. pyogenes*, and *E. faecalis*), two strains were Gram-negative bacilli (*E. coli* and *P. aeruginosa*), whereas two strains were represented by yeasts (*C. albicans* and *C. parapsilosis*).

MICs of the tested OEO were similar to those obtained for OEO-PbH, lower (2.5 µg/mL) for yeast, and higher (40–80 µg/mL) for Gram-negative bacilli (Table 4).

### 3.5. The Antiproliferative/Cytotoxic Activity of OEO-PbH

As keratinocytes are among main components of skin tags, an in vitro evaluation was conducted in order to see the effect of the modern formulation against HaCaT human keratinocytes.

#### 3.5.1. MTT Assay

**Figure 4:**
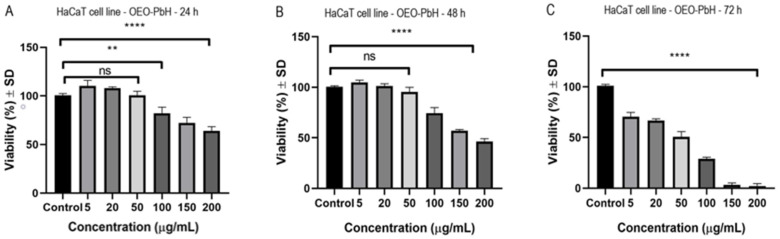
HaCaT cell viability at (**A**) 24 h, (**B**) 48 h, and (**C**) 72 h after stimulation with different concentrations of OEO-PbH (5, 20, 50, 100, 150, 200 µg/mL). Data are represented as mean ± SD. Comparison among groups was made with one-way ANOVA and Dunnett’s multiple comparison test (** *p* < 0.01, **** *p* < 0.0001 vs. Control; ns = not significant).

The effect of OEO-PbH was evaluated at different time periods on HaCaT human keratinocytes. The control represents cells treated with cell culture media. In Figure 4A is depicted the effect of OEO-PbH at 24 h post-stimulation. It can be observed that at concentrations ranging from 5 to 50 µg/mL there is a slight increase in cell viability. By increasing the concentration, a dose-dependent decrease in HaCaT viability was noticed. In Figure 4B is represented the effect at 48 h post-stimulation. At 5 µg/mL and 20 µg/mL, HaCaT viability was not affected following treatment with OEO-PbH (at 5 µg/mL cells viability was 104.7 ± 2.3% and at 20 µg/mL was 101.1 ± 2.2% vs. Control). At concentrations above 50 µg/mL a decrease in cell viability was obtained. Figure 4C depicts the effect of OEO-PbH at 72 h post-stimulation. At all the tested doses a reduction in keratinocyte viability was observed. At the lowest dose tested (5 µg/mL), cell viability was 70.3 ± 4.3% vs. Control and at the highest dose tested (200 µg/mL), HaCaT viability was 2 ± 2.5% vs. Control. HaCaT viability was not affected by the stimulation with B-PbH at any tested concentration (5, 20, 50, 100, 150, and 200 µg/mL). Table 5 shows the IC_50_ (half-maximal inhibitory concentration) values of OEO-PbH for different time periods.

#### 3.5.2. Scratch Assay

**Figure 5:**
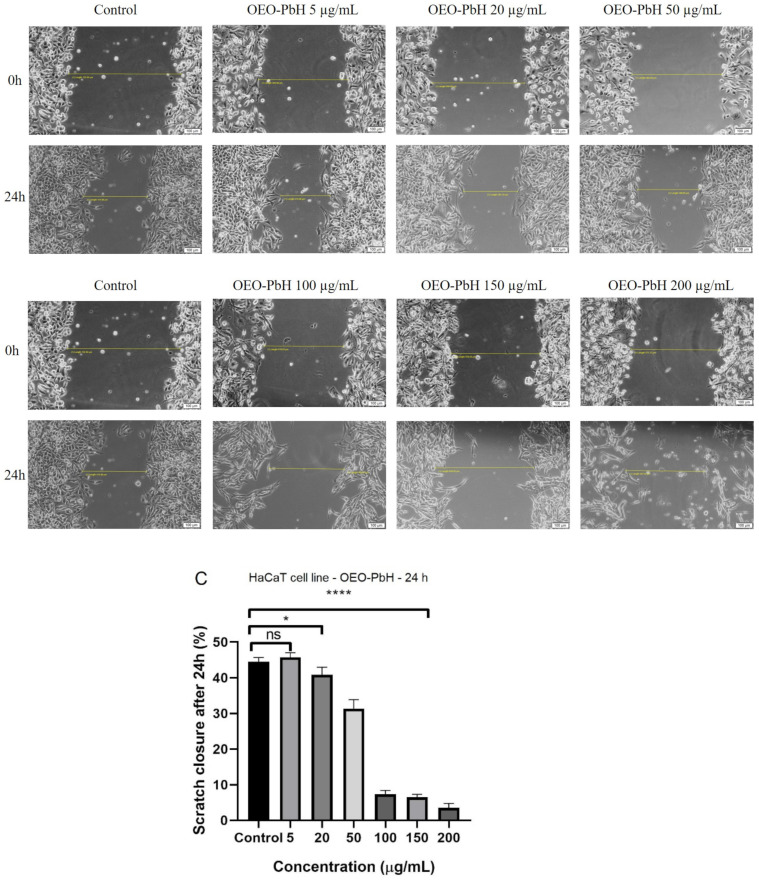
OEO-PbH (**A**) (5, 20, and 50 μg/mL) and (**B**) (100, 150, and 200 μg/mL) effect on HaCaT human keratinocytes migration and proliferation potential. (**C**) Scratch closure rate after 24 h. Progression of cell migration was evaluated by imaging the scratch line at 0 h and at 24 h post-stimulation. Images were taken by light microscopy at 10× magnification. Comparison among groups was made with one-way ANOVA and Dunnett’s multiple comparison test (* *p* < 0.05, **** *p* < 0.0001 vs. Control; ns = not significant).

The anti-migratory effect of OEO-PbH at different concentrations was determined using a scratch assay. The data from this study (Figure 5) showed that following application of the samples, a dose-dependent decrease in keratinocytes migration was observed. As shown in Figure 5C, a significant decrease in HaCaT migration was observed at concentrations higher than 100 µg/mL.

#### 3.5.3. LDH Assay

**Figure 6:**
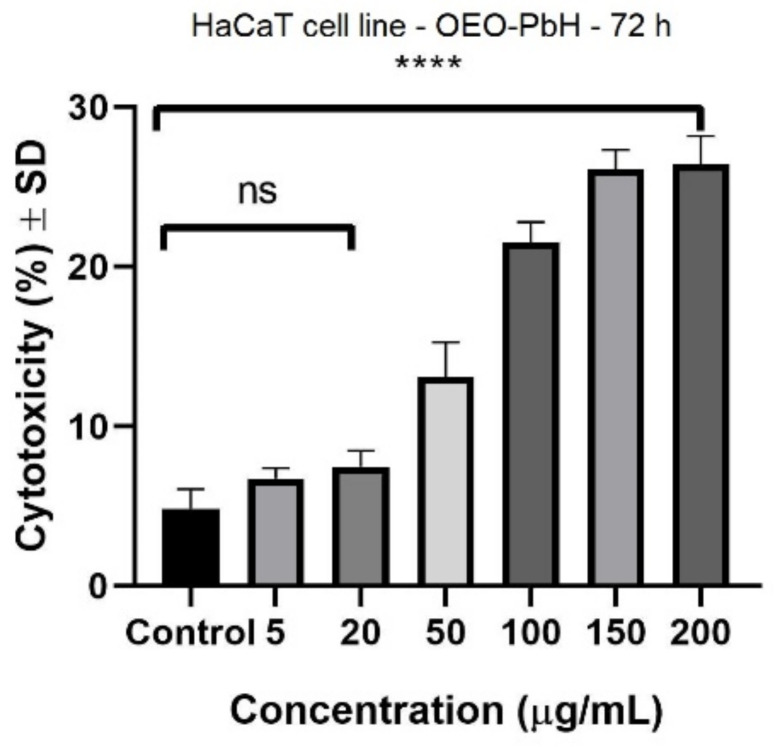
The cytotoxicity of different concentrations of OEO-PbH (5, 20, 50, 100, 150, 200 µg/mL) at 72 h post-stimulation. Data are represented as mean ± SD. Comparison among groups was made with one-way ANOVA and Dunnett’s multiple comparison test (**** *p* < 0.0001 vs. Control; ns = not significant).

Figure 6 presents the cytotoxic effect of OEO-PbH at 72 h after stimulation based on LDH release. It can be noticed that starting from 50 µg/mL there is an increase in LDH release. At the highest tested concentrations, a significant cytotoxic effect was elicited at 150 µg/mL (26.1 ± 1.2%) and at 200 µg/mL (26.4 ± 1.7%) vs. Control (4.8 ± 1.2%). B-PbH did not show any cytotoxic effects against the HaCaT cell line at the tested concentrations (5, 20, 50, 100, 150, and 200 µg/mL).

#### 3.5.4. Nuclear Staining

**Figure 7:**
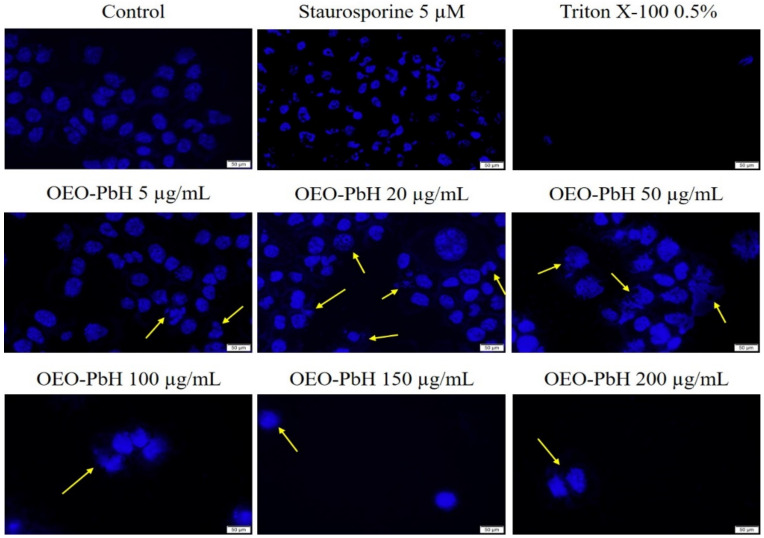
Hoechst staining in HaCaT cell line following OEO-PbH (5, 20, 50, 100, 150, 200 µg/mL) stimulation (72 h). Staurosporine (5 µM) was used as an indicator for apoptosis induction and Triton X-100 (0.5%) for necrosis. The scale bar represents 50 µm.

In order to obtain a more detailed insight on the pro-apoptotic effect of the sample on cell nuclei, Hoechst staining was performed. As depicted in Figure 7, the Control group (untreated) has round nuclei with regular shape. Stimulation with OEO-PbH induced changes in cell nuclei—fragmentation and membrane blebbing. The most important features of the pro-apoptotic effect are represented by yellow arrows. Moreover, a reduced number of cell nuclei can be observed by increasing the concentration of the sample.

### 3.6. Immunomodulatory Effects on Human DCs

**Figure 8:**
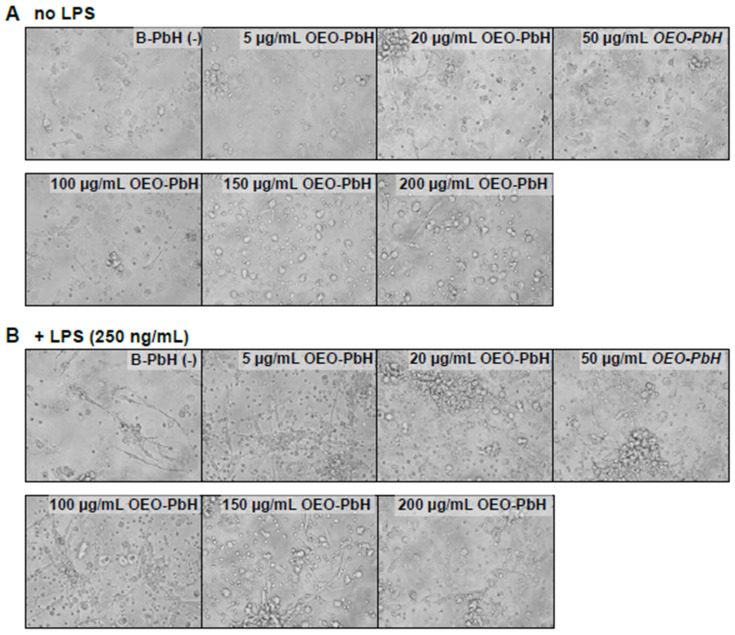
Brightfield microscopy images of OEO-PbH treated human DCs. DCs were cultured for 7 days with GM-CSF and IL-4. Cells were subsequently stimulated without LPS (**A**) or with LPS (**B**) and treated with either OEO-PbH or B-PbH formulations at different concentrations for 24 h. The images were taken under a light microscope using a 20× objective.

In-vitro-generated DCs (+ GM-CSF, + IL-4) cultured from blood-derived human PBMCs were stimulated with the OEO-PbH formulation in order assess effects on cell viability or cytokine secretion. To this end, we analyzed the induction of apoptosis or viability of human DCs 24 h after treatment with OEO-PbH in either naïve (no LPS) DCs or under inflammatory conditions (together with LPS). First of all, representative bright-field microscopy images revealed no obvious effect on the viability and cell morphology of in-vitro-generated DCs compared to B-PbH-treated cells (Figure 8).

**Figure 9:**
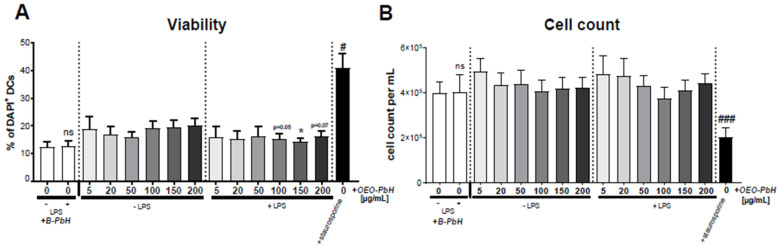
Effects of OEO-PbH on the viability of human DCs. DCs were cultured for 7 days with GM-CSF and IL-4. Cells were subsequently stimulated without LPS or with LPS and treated with the OEO-PbH at different concentrations for 24 h. Viability (**A**) as well as the absolute number of DCs (**B**) were assessed using flow cytometry. Staurosporine-treated DCs served as a positive control (black bar). (**A**) Frequency of DAPI^+^ (dead cells). (**B**) Absolute cell number in cell culture 24 h after stimulation calculated as cells/mL. Data expressed as mean ± SEM (*n* = 6 donors); # indicates comparisons to the respective blank control (B-PbH-treated DCs) using RM one-way ANOVA; * indicates group comparisons between LPS-treated and LPS-untreated cells using paired *t*-test (# *p* < 0.05; ### *p* < 0.001; ns = not significant).

Analysis of DAPI staining, which stains dead cells, further showed no cytotoxic effect of OEO-PbH (Figure 9A) and no effects on the absolute numbers of DCs (Figure 9B). Interestingly, LPS-treated DCs that were additionally treated together with higher concentrations of OEO-PbH (100–200 µg/mL) appeared to have a reduced frequency of DAPI^+^ cells.

**Figure 10:**
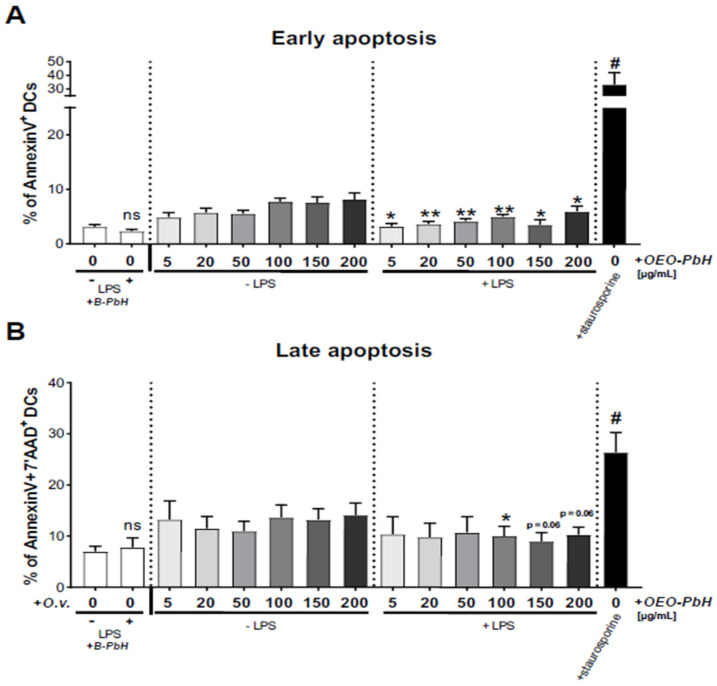
Effects of OEO-PbH on the apoptosis of human DCs. DCs were cultured for 7 days with GM-CSF and IL-4. Cells were subsequently stimulated without LPS or with LPS and treated with OEO-PbH at different concentrations for 24 h. The frequency of early (AnnexinV^+^) apoptotic DCs (**A**) as well as late (AnnexinV^+^/7′AAD^+^) apoptotic DCs (**B**). Staurosporine-treated DCs were used as a positive control (black bar). Data expressed as mean ± SEM (*n* = 6 donors); # indicates comparisons to the blank control (B-PbH-treated DCs) using RM one-way ANOVA; * indicates group comparisons between LPS-treated and LPS-untreated cells using paired *t*-test (* *p* < 0.05; ** *p* < 0.01; ns = not significant).

Despite no obvious cytotoxic effects of the OEO-PbH formulation and as the treatment may affect the apoptosis, subsequently the induction of early (AnnexinV^+^) and late apoptosis (AnnexinV^+^/7′AAD^+^) in DCs was analyzed (Figure 10). Surprisingly, the treatment with OEO-PbH in inflammatory DCs significantly lowered the induction of especially early apoptosis (Figure 10A). Further, it can be observed also a tendency of reduced late apoptotic DCs after the treatment with higher concentrations (100–200 µg/mL) of OEO-PbH in inflammatory DCs (Figure 10B).

**Figure 11:**
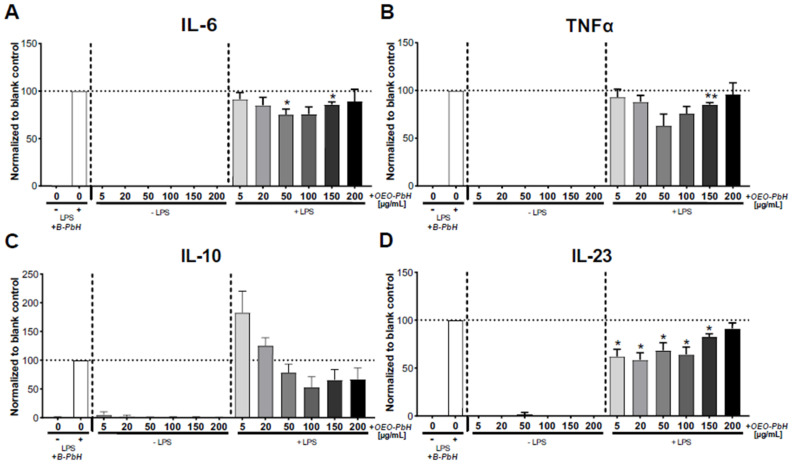
Effects of OEO-PbH on the cytokine production. DCs were cultured for 7 days with GM-CSF and IL-4. Cells were subsequently stimulated without LPS or with LPS and treated with OEO-PbH at different concentrations for 24 h. The DC cell culture supernatant was then analyzed by ELISA for IL-6 (**A**), TNFα (**B**), IL-10 (**C**), and IL-23 (**D**). Data expressed as mean ± SEM (*n* = 6 donors); * indicates comparisons to the respective blank control (B-PbH-treated DCs) using RM one-way ANOVA (* *p* < 0.05; ** *p* < 0.01).

As we found no harmful effect on the viability of DCs, we were interested to study cytokine secretion also in order to see if the OEO-PbH formulation might modulate the release of cytokines, such as IL-6, IL-10, IL-23, and TNF-α, and whether this might implicate potential effects of this formulation on the immune response of human DCs. Importantly, the treatment with OEO-PbH was not able to stimulate the cytokine production in naïve (LPS-untreated) DCs (Figure 11). Interestingly, OEO-PbH treatment in inflammatory DCs (+ LPS) indicated a decrease in cytokine production of IL-6 (Figure 11A), TNF-α (Figure 11B), and IL-23 (Figure 11D) but no significant effect on IL-10 (Figure 11C) in any of the tested concentrations compared to B-PbH-treated cells.

## 4. Discussion

According to the scientific literature, the main active constituents of OEO are terpenes (carvacrol and thymol) [5]. In the screened sample in this study, 43 compounds were identified with carvacrol and thymol as main compounds, as depicted in the results section. In terms of biological activities, the antibacterial, antiparasitic, antifungal, and insecticidal activities of OEO are due to the synergistic effects of the multitude of components present in the volatile oil (e.g., carvacrol, thymol, α-terpinene, γ-terpinene, β-linalool, β-myrcene) [50,51,52]. Moreover, besides the proved biological properties of carvacrol and thymol, compounds such as caryophyllene and *p*-cymene are also of great interest for their anti-inflammatory, antioxidant, and antiproliferative activities [53,54].

Rodrigues et al. pointed out that a higher amount of carvacrol instead of thymol is habitually reported in the literature [55]. It is well known that the volatile oil composition varies widely according to the plant origins and growth conditions [8]. Employing hydro distillation as extraction method and GC-MS analysis as compound detection method, Figiel et al. have found that the major compounds in Polish OEO are carvacrol (3.6–9.1 g/kg) and thymol (2.14–8.44 g/kg) [56]. On the other hand, Ruben et al. observed that the major components in Argentine OEO were γ-terpinene (25.1%), terpineol-4-ol (16.7%), carvacrol (16.2%) and α-terpinene (8.54%) [57]. Khan et al. showed that Saudi oregano contains carvacrol (70.2 ± 1.37%) as the main component, while Jordanian oregano has thymol as the major compound (68.7%) [58]. According to De Martino et al., high content of carvacrol and thymol (21.89 ± 0.70% and 18.21 ± 0.80% respectively) has been also determined for oregano volatile oil from Italy [59]. Serbian OEO is also characterized by a high amount of carvacrol (72.06%) and thymol (4.98%), pursuant to Karaman et al. [60]. Teixeira et al. analyzed the composition of OEO collected from Portugal by the GC-MS method and discovered a substantial quantity of oxygenated monoterpenes (53.8%), among which the major compounds were carvacrol (14.5%) and thymol (12.6%) [61]. Garcia-Perez et al. managed to show that carvacrol and thymol were the two terpenes represented in higher amount in Mexican OEO (29.99% and 4.51%, respectively) [62], while Tapiero et al. exposed that the main components of the evaluated samples were *p*-cymene (10.1%), terpinen-4-ol (9.48%), carvacrol (8.90%), and thymol (1.95%) [63]. In order to characterize the composition of OEO obtained from plants collected in Brazil, Viana Dutra et al. established that carvacrol acetate was the major compound identified, in a percentage of 69.51% [64]. On the other hand, Hashemi et al. identified thymol as the main compound (32%), followed by 4-terpineol (12%), α-terpinene (10%), and carvacrol (6%) in OEO harvested from Iran [65]. Mechergui et al. studied the effect of harvest year on chemical composition and antioxidant activity of OEO collected from the north of Tunisia and concluded that, despite the variations that took place, the main compounds remained *p*-cymene, γ-terpinene, thymol, and carvacrol [66]. Alinkina et al. also studied the composition of this aromatic plant and concluded that the total amount of phenolic compounds of industrial OEO comprises a percentage of 67.51% (carvacrol—63.28%, thymol—4.23%) [67].

It is well known that due to their hydroxyl groups, phenolic compounds demonstrate a potent antioxidant activity, interrupting oxidation reactions by acting as an acceptor of free radicals [68,69]. The antioxidant activity of OEO is also related to the high amount of two phenolic monoterpenes, carvacrol and thymol [70]. Because of the fact that the results may depend on the method used, it is recommended to use at least two methods when evaluating the antioxidant activity of a volatile oil [71]. To investigate the antioxidant capacity of OEO, the following methods were employed: DPPH, ABTS, CUPRAC, and ORAC. As described in the results section, the CUPRAC method recorded the highest concentration of equivalent Trolox, showing a value of 5320.1 ± 32 TE µmol/mL, followed by ORAC method with a value of 3752.7 ± 28 TE µmol/mL. The tested sample showed a value of 1304 ± 20 TE µmol/mL when the ABTS assay was employed. Complementarily, the DPPH method indicated a scavenging activity of 86 ± 28%. These data are proof of the antioxidant action of OEO obtained from the western part of Romania. In the same line, screening the antioxidant activity, Ghadermazi et al. evaluated the radical scavenging activity of OEO procured from Iran by using the DPPH method. The research group obtained an amount of 57.89 ± 2.05% for a concentration of 1000 µg/mL methanolic solution of essential oil [72]. Using the same method, Viuda-Martos et al. reported that the antioxidant action of Spanish oregano essential oil at 5 g/L concentration was 51.79%, while at a concentration of 50 g/L the inhibition increased to 87.19% [73]. Gavaric et al. showed that Serbian OEO possessed stronger antioxidant activity than *terc*-butylated hydroxytoluene, used as positive control [74]. Tapiero et al. determined the antioxidant activity for two different samples of OEO acquired from Colombia by DPPH (310.8–320.6 mmol Trolox/100 g) and ABTS (23.128–24.019 mmol Trolox/100 g) [63]. Pursuant to Garcia-Perez et al., the highest antioxidant activity of oregano oil was obtained through the ABTS method (35.8 ± 0.4 µmol TE/g extract), followed by DPPH assay with 6.1 ± 0.4 µmol TE/g extract [62]. In order to analyze the antioxidant activity of OEO, Viana Dutra et al. applied the DPPH and ABTS methods and obtained the following values: 363 µmol Trolox/mg, and 1142 µmol Trolox/mg, respectively [64]. Ruben et al. determined the radical scavenging activity of oregano oil by DPPH-FRSA test and obtained an amount of 84.1% [57]. The DPPH assay used by Mechergui et al. yielded a variable antioxidant range for OEO during the harvest years, namely between 59.2 mg/L and 226.19 mg/L [66]. Employing the ORAC assay, Beirão et al. obtained a value of 2080 ± 21 µmol TEAC/g for Portuguese OEO used in encapsulation process [75]. In the same line, Asensio et al. concluded that OEO collected from Rio Negro, Argentina exhibited an increased ORAC value (1.708 TE) [76], while Bentayeb et al. obtained a value of 2.25 g Trolox/g essential oil for OEO purchased from Spain [77]. Anastasiou et al. studied the antioxidant activity of Greek OEO. Therefore, the highest value was obtained through the ABTS method (5879 ± 325 µmol/mL), followed by the ORAC method (4147 ± 578 µmol/mL), while the CUPRAC assay delivered the lowest value (1441 ± 271 µmol/mL) [37].

Considering the lipophilic nature of the OEO, one of the well-known challenges for formulating a topical hydrophilic semisolid system is its solubilization without using a cosolvent (i.e., ethanol or isopropanol) which can be a skin irritant. Therefore, by selecting a polymeric micelles system, the micellar solubilization of OEO can be achieved and also the OEO-loaded polymeric micelles can act as colloidal carriers of the active ingredient into the skin, improving/promoting its dermal penetration. Moreover, OEO polymeric micelles systems based on poloxamers are expected to be biocompatible and safe for the skin (non-irritant and non-toxic). All the above mentioned attributes support the superiority of polymeric micelles systems for OEO dermal delivery over other hydrophilic conventional dosage forms (i.e., hydrogels and hydrophilic creams).

Regarding the physicochemical characterization of the designed formulation, OEO-PbH, the pH values of both OEO-PbH and B-PbH were in accordance with the nonionic character of Pluronic F 127 and L 31 and with previously reported pH values of poloxamer 407 aqueous solutions (very close to neutral) [78]. Similarly, a 20% poloxamer 407/10% poloxamer 188 gel formulation presented a pH value closely to neutral (6.52 ± 0.06) [79]. The pH values fall within the accepted range for skin application (4.5–8) [80] and reveal the poloxamer binary hydrogels compatibility with the skin, a key aspect for patient acceptance. At the same time, a yellowish color characterizes the appearance of topical formulations containing OEO, while the transparency and homogeneity remain unchanged [81]. Among the various rheological properties currently investigated for rheological characterization of semisolid formulations, the consistency was of interest for this study as it can quantify their ease of application to the skin (through the spreadability) and prolonged residence time on the treated area (through the penetration value), two key features for patient acceptability and improved therapeutic efficacy, respectively [41].

The values of the spreading area produced by both formulations increased progressively with the increase of applied standardized weight, indicating they are relatively easily spreadable systems. Further, it can be noticed that for all the applied weights, OEO-PbH showed significantly smaller spreading areas than that produced by B-PbH, which proved its higher consistency. As it was expected, these observations confirmed the results of the penetrometric test, suggesting the formation of consistent semisolid poloxamer binary hydrogels, with ordered packing of polymeric micelles due to considerable intermicellar interactions at a 20% concentration of poloxamer 407. Additionally, the increase in consistency determined by the incorporation of OEO in the blank formulation can be attributed to the effect of the essential oil on the micellization and gelation processes of the poloxamers.

Colloidal solutions and suspensions are essential for life—they work in everyone’s cells, blood, and body fluids, especially intercellular ones. The determination of particle size and zeta potentials is based on the dynamic light scattering or photon correlation spectroscopy [82]. The important difference between the values of zeta potentials is very interesting: they indicate that B-PbH has a medium tendency to form clusters based on the description from the literature [83], while the OEO-PbH has an increased tendency to agglomerate.

The antimicrobial activity of essential oils is well known, with a mechanism that involves affecting the cytoplasmic membrane of microorganisms with impeding transport systems and energy production. However, it must be specified that according to the composition of the walls, the Gram-positive bacteria and yeasts led to slightly lower MIC compared to Gram-negative bacilli. In Gram-negative bacteria, the antibacterial effect is conditioned by the penetration of the compound through the porins in the outer membrane, which causes the MIC to be increased or the antibacterial effect to be slowed down compared to that expressed on Gram-positive bacteria or *Candida* spp [84]. MBC and MFC were equal to MIC for both OEO and OEO-PbH. The MIC values reported by other authors vary greatly, between 0.5 and 250 × 10^3^ µg/mL [14,59,85,86,87], with our results (2.5–80 µg/mL) being included in this interval. Therefore, it can be concluded that the antimicrobial activity of OEO is preserved after incorporation in the poloxamer mixture. Rosato et al. showed that OEO (acquired from Corato, Bari, Italy), having thymol and carvacrol as main active compounds (59.25% and 25.09%, respectively), presented effective antimicrobial and anti-biofilm activities against different strains of Gram-positive bacteria. OEO exhibited MIC values of 5.5 × 10^3^ µg/mL on *E. faecalis* ATCC 29212, 5.8 × 10^3^ µg/mL on *S. aureus* ATCC 29213 and 11.0 × 10^3^ µg/mL on *S. aureus* Ig22 [88]. In order to investigate the potential of OEO (purchased from Zagreb, Croatia) to be used as an antimicrobial agent in gynecological bacterial and fungal infections, Karaman et al. determined MIC, MBC, and MFC values of 12 bacteria strains and three yeast strains. The chemical composition of OEO is characterized by a high amount of carvacrol (72.06%) and thymol (4.98%). The most susceptible bacteria were *E. coli* 1 strain (MIC = 1.4 × 10^3^ µg/mL, MBC = 2.8 × 10^3^ µg/mL) and *S. aureus* 3 strain (MIC = 11.4 × 10^3^ µg/mL, MBC = 22.7 × 10^3^ µg/mL). At the same time, filament formation of *C. albicans* was completely inhibited at an MFC value of 4.5 × 10^3^ µg/mL OEO [60]. Likewise, Italian OEO (47.31% *p*-thymol) presented great antibacterial effect against *E. coli* JM109 strain (MIC = 300 µg/mL) [89]. Moreover, Ebani et al. determined that OEO provided from FLORA^®^, Pisa, Italy, characterized by a high level of carvacrol (65.9%), showed remarkable antimicrobial activity against bacteria strains involved commonly in urinary tract infections (*E. coli* and *Enterococcus* spp.), with MIC values ranging from 0.293 × 10^3^ µg/mL to 1.183 × 10^3^ µg/mL [90]. On the contrary, according to Fournomiti et al., *E. coli* strains were the most resistant to Greek OEO, regardless of the cultivation conditions, exhibiting significantly increased MIC values (MIC = 236.1 × 10^3^ µg/mL for irrigated oregano crops and MIC = 219.9 × 10^3^ µg/mL for non-irrigated oregano crops) [14]. The Canadian OEO showed effective antimicrobial and inhibitory activities, providing equal MIC and MBC values on *S. pyogenes* ATCC 19615 and ATCC 49399 strains (0.5 × 10^3^ µg/mL) [91]. La Pergola et al. analyzed the relationship between antibacterial effect of commercially available and wild Sicilian OEO and their chemical composition. According to GC-MS results, the main compounds of commercial OEO were thymol and γ-terpinene (48.7% and 20.5%, respectively), while wild OEO contained as the major component 66.1% carvacrol. Although both tested OEO exhibited an MIC value of 3 µg/mL against *E. coli* 10198 strain, a significant statistical difference (*p* ≤ 0.01) was observed between MIC values against *S. aureus* 1104 strain (MIC = 3 µg/mL for wild OEO; MIC = 4 µg/mL for commercial OEO) [92]. Algerian OEO (41.6–81.1% thymol as major compound, depending on the extraction method) showed a stronger antifungal effect against *C. albicans* strains (MIC values from 36 µg/mL to 57 µg/mL) than the antimicrobial activity against *S. aureus*, *E. coli* and *P. aeruginosa* (MIC ranging from 64 µg/mL to 120 µg/mL) [93]. Another study revealed that the encapsulation process of OEO (cultivated in Pazardzhik, Bulgaria) in chitosan–alginate nanoparticles improved the antimicrobial effect of the essential oil. The pure oil presented MIC values ranging between 0.0625 and 1% against *S. aureus*, *E. faecalis* and *E. coli*—0.0625%, *C. albicans*—0.125%, and *P. aeruginosa*—1%. On the other hand, the nanoformulation exhibited significantly increased inhibitory effect against the tested strains, with MIC values of 0.0078% against *S. aureus*, *E. faecalis*, *E. coli*, 0.0156% against *C. albicans,* and 0.25% against *P. aeruginosa* [94]. Similarly, Kozics et al. demonstrated that OEO was effective in preventing the growth of drug-resistant microorganisms responsible for wound infections, including *P. aeruginosa*, *Proteus vulgaris* (*P. vulgaris*), *Citrobacter koseri* (*C. koseri*), and *Klebsiella pneumoniae* (*K. pneumoniae*) but also *C. albicans* and *C. parapsilosis*. OEO showed significantly strong antibacterial and antifungal activities against those strains, with MIC 0.025–0.125% against multidrug resistant bacteria and MIC 0.05% against *Candida* strains [95]. Man et al. examined the antimicrobial behavior of two solutions of OEO (80.5% carvacrol), obtained by means of broth microdilution, against the most common pathogens. The first solution contained micelle aggregates of OEO, while the second one included the hydrosoluble components of OEO obtained by aqueous extraction. Micellar extract showed the best inhibitory effect against methicillin-sensitive *S. aureus*, methicillin-resistant *S. aureus*, *E. faecalis*, *E. coli*, and *P. aeruginosa* (MIC ranging from 0.1% to 6.3%), while the aqueous solution presented lower inhibitory effect (MIC ranging from 12.5% to 25%) [96].

Skin tags, known also under the name of acrochordons or fibroepithelial polyps are the most common benign skin tumor, with a prevalence of 50–60% in the adult population [97]. These papillomas consist of epidermal tissue connected with a thin stalk to the skin and present a size of 1 mm–1 cm. Because of the fact that they occur in the skin folds, skin tags can be very bothersome for the patients, mostly due to aesthetic reasons and irritation symptoms caused by friction [98,99,100]. The nature of the composition of skin tags (keratinocytes, small blood vessels) and the lack of a non-invasive method of removal up to date led us to the idea of conception, characterization and evaluation of an innovative OEO poloxamer based hydrogel as a possible candidate for the management of this very common skin problem. As a first step in the comprehensive evaluation, the study aims to test the antiproliferative and pro-apoptotic potential in vitro against HaCaT human keratinocyte cell line.

The viability of HaCaT was assessed after exposure to OEO-PbH. After 24 h and 48 h post-stimulation, the cell viability decreased in a dose-dependent manner after exposure to concentrations of 100 µg/mL and above, while the exposure to all the tested concentrations leaded to a marked decrease of keratinocytes viability after 72 h post-stimulation. The decrease in cell viability is needed in order to obtain the expected effect of OEO-PbH which is represented by the drying and falling of papillomas. In a similar approach, Avola et al. established that OEO (obtained from Milan, Italy), having carvacrol and thymol as main phytochemicals, showed non-significant decrease in cell viability against human keratinocytes (NCTC 2544 cell line) at the working concentrations (3, 5, 7.5, 10, 12, 25, and 50 µg/mL) [101]. In the same vein, Laothaweerungsawat et al. remarked that 5% OEO (collected from Chaing Mai, Thailand), having carvacrol as main component, and 5% carvacrol (acquired from St. Louis, MO, USA) did not affect the human keratinocytes HaCaT cells viability (>96%) at the working concentrations (3.125–50 µg/mL). On the other hand, the 5% OEO microemulsion decreased HaCaT viability to 71.7 ± 6.6%, possibly due to other components in the microemulsion (Tween 60 and/or butylene glycol) [81]. Therefore, the results obtained by our research team are consistent with those obtained in the aforementioned studies since a decrease in cell viability was observed at concentrations above 100 µg/mL OEO-PbH. On the other hand, the results obtained in the following studies show that pure OEO, i.e., carvacrol and thymol, exhibit a strong cytotoxic effect at low concentrations (lower than our tested concentrations). The cytotoxic effect of OEO (purchased from Pleasant Grove, UT, USA) on HaCaT cell line, having carvacrol and thymol as main active substances, was evaluated by Kozics et al. by means of MTT assay. After 24 h of exposure to different concentrations, the viability decreased dose-dependently, and the IC_50_ value for OEO was 0.018% [95]. Similarly, the cytotoxicity analysis of OEO and poly(ε-caprolactone) encapsulated OEO, provided from Milan, Italy, on HaCaT cells revealed IC_50_ values of 0.093 mg/mL and 0.044 mg/mL, respectively [102]. Yoncheva et al. observed that OEO (collected from Pazardzhik, Bulgaria) exhibited increased cytotoxic activity against HaCaT cells compared to chitosan–alginate encapsulated OEO (IC_50_ = 0.00631% and 0.0255%, respectively) [94]. Using HaCaT cells as control line, Rojo-Ruvalcaba et al. determined that 0.028 M carvacrol (acquired from St. Louis, MO, USA) led to a reduction of the proliferation index value of 33.06 ± 5.35%, while 0.042 M and 0.056 M determined the death of approximate 90–100% of the cells [103]. Following the Blue Cell viability assay, García-Salinas et al. also assessed the antiproliferative activity of carvacrol and thymol (purchased from St. Louis, MO, USA). Doses of 0.090 mg/mL and higher of thymol and carvacrol decreased human dermal fibroblast cells viability under 40%. On the other hand, 0.060 mg/mL thymol and 0.030 mg/mL carvacrol induced subcytotoxic effects on HaCaT cells [104]. Applying the same method mentioned above, Yao et al. studied the cytotoxic potential of OEO and carvacrol (provided from Nanning, Guangxi, China), the main active compound, against human foreskin fibroblast (HFF) cell line. MTT analysis pointed out that 134.9 µg/mL OEO produced the death of 50% HFF cells, while the same percentage was achieved by 43.93 µg/mL carvacrol [105]. Moreover, the cytotoxic effects of carvacrol and thymol obtained from St. Louis, MO, USA, against human fibroblasts cell line (WS-1) was also studied by Günes-Bayir et al. by means of ATP cell viability assay. The cell viability was considerable reduced in a concentration-dependent manner with IC_50_ values of 138.1 ± 8.7 µM for carvacrol and 167 ± 11 µM for thymol [106,107]. Commercially available OEO (dõTERRA, Pleasant Grove, UT, USA), containing a high amount of carvacrol, was evaluated on human dermal fibroblast cell line by sulforhodamine assay. Researchers observed that the tested product was found to be toxic at a concentration of 0.011% [13].

OEO-PbH presented a cytotoxic effect starting from 50 µg/mL, while the highest tested concentrations (150 µg/mL and 200 µg/mL) led to an important amount of LDH release and a significant cytotoxic effect against HaCaT cell line, respectively. Janani et al. measured the lactate dehydrogenase release of L929 fibroblast cell line following incubation with 50–400 µg/mL OEO procured from Tamil Nadu, India. The amount of LDH release was insignificant at 50 µg/mL OEO, while fibroblasts showed an important loss of cell wall integrity at 100–400 µg/mL OEO [108]. In the same line, Ranjitkar et al., using CCD-1123Sk human skin fibroblasts, observed that carvacrol determined important damages of membrane integrity starting from 100 µg/mL [109].

Scratch assays revealed that OEO-PbH decreased cell migration in a dose-dependent manner and concentrations higher than 100 µg/mL, leading to a significant decrease in HaCaT migration.

The apoptotic effect of thymol and carvacrol on human fibroblast (WS-1) cell line was studied employing acridine orange/ethidium bromide staining assay. Doses of 10–100 µM thymol/carvacrol decreased in a concentration-dependent manner the cell viability, while the amount of apoptotic and necrotic cells increased significantly. The microscopic images showed characteristic morphological changes, such as chromatin condensation and cell contraction. Western blot analysis revealed that both compounds progressively increased the amount of pro-apoptotic proteins (caspase-3, caspase-9, Bax), while the levels of anti-apoptotic protein Bcl-2 were reduced [106,107].

In this comprehensive evaluation we also wanted to check the effect on the immune cells. Data on the effects of the OEO-PbH formulation in human DCs showed no cytotoxic effect. The treatment with OEO-PbH in inflammatory DCs rather indicated an even reduced induction of early apoptosis in inflammatory DCs. This further strengthens the observation that OEO-PbH has no harmful effect in the tested concentration range between 5 and 200 µg/mL on the cell morphology and viability. Moreover, the DCs showed reduced levels of IL-6 and TNF-α in OEO-PbH-treated inflammatory DCs. Since both pro-inflammatory cytokines are considered as targets for therapy in autoimmune diseases, such as rheumatoid arthritis [110], our data provide an indication for an anti-inflammatory effect of this formulation. IL-23, which is known to drive Th17-related immune responses and which is involved in the pathogenesis of psoriasis [111,112], was found to be additionally reduced in OEO-PbH-treated inflammatory DCs. Thus, the application of this pharmaceutical formulation may dampen the immune responses under chronic inflammatory conditions. Since our results in vitro showed that OEO-PbH does not drive the immunomodulation in absence of any inflammatory stimulus, the active ingredient seemed to interfere or counteract the inflammatory stimulation with LPS. In summary, the results presented in the present study highlight that the OEO-PbH formulation might have potential immunotherapeutic value to limit the inflammatory response in human DCs.

## 5. Conclusions

OEO collected from the western part of Romania contains carvacrol and thymol as the main compounds and presents antioxidant activity. A Pluronic F 127/L 31 binary hydrogel loaded with oregano essential oil (5% *w*/*w*) was successfully prepared and its physicochemical properties were in accordance with the specific requirements for semisolid pharmaceutical preparations. Among the tested strains, both the oil as well as the formulation presented lower antimycotic effect (2.5 µg/mL for yeast) and higher antibacterial (40–80 µg/mL for Gram-negative bacilli) potential. HaCaT cell proliferation and migration were inhibited by the hydrogel formulation at concentrations of 100 μg/mL and above, while the cytotoxicity of the hydrogel was emphasized by a strong LDH release. OEO-PbH also induced changes in morphology and quantity of cell nuclei, exhibiting a pro-apoptotic effect. The data indicates that OEO-PbH elicited a significant dose-dependent effect against HaCaT cells. Whereas skin tags are composed by keratinocytes and small blood vessels, the antiproliferative, antimigratory, cytotoxic, and pro-apoptotic effects of OEO-PbH may lead to the drying and falling off of the skin tags. Moreover, the formulation might have potential immunotherapeutic value to limit the inflammatory response in human DCs. The in vitro results obtained in this study are encouraging for the evaluation of the in vivo effect.

## Figures and Tables

**Table 1:** Identified compounds by GC-MS.

Compound	*R*_t_ (min)	Concentration (% of Total Peak Areas)	Molecular Weight (g/mol)	Molecular Structure
α-thujene	7.697	0.11	136	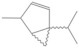
α-pinene	7.946	0.49	136	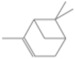
Camphene	8.518	0.15	136	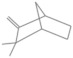
β-pinene	9.498	0.06	136	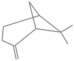
1-octen-3-ol	9.578	0.11	128	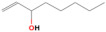
β-myrcene	9.911	1.06	136	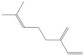
α-phellandrene	10.54	0.19	136	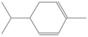
n.i.	10.616	0.06		
α-terpinene	10.927	1.12	136	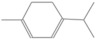
*p*-cymene	11.221	4.13	134	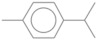
D-limonene	11.388	0.16	136	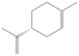
β-phellandrene	11.454	0.19	136	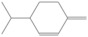
Eucalyptol	11.515	0.19	154	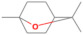
Trans-β-ocimene *	11.653	0.02	136	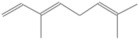
Cis-β-ocimene	12.055	0.05	136	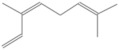
γ-terpinene	12.498	4.08	136	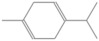
n.i.	12.967	0.21		
Terpinolene	13.536	0.16	136	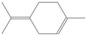
Benzene, (2-methyl-1-propenyl)-	13.737	0.05	132	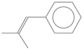
β-linalool	14.113	6.46	154	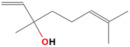
n.i.	15.075	0.02		
α-campholenal *	15.151	0.02	152	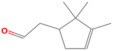
n.i.	15.687	0.01		
n.i.	15.763	0.01		
Camphor	15.951	0.03	152	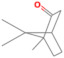
Borneol	16.897	0.99	154	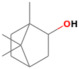
Terpinen-4-ol	17.219	0.97	154	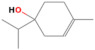
n.i.	17.473	0.03		
α-terpineol	17.8	0.27	154	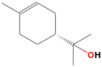
Thymol methyl eter	19.373	0.2	164	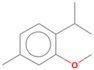
Carvone	19.679	0.13	150	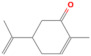
Linalool acetate	19.818	0.04	196	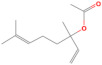
Carvenone	20.261	0.02	152	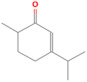
Thymol	21.264	1.7	150	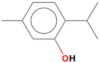
Carvacrol	21.691	71.21	150	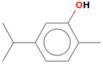
n.i.	22.171	0.14		
Thymol acetate	23.915	0.15	192	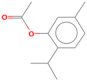
Methyleugenol	25.151	0.02	178	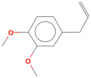
Caryophyllene	25.867	1.36	204	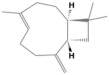
α-bergamotene *	26.286	0.03	204	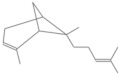
Aromadendrene *	26.491	0.17	204	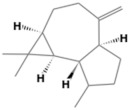
α-caryophyllene	27.079	0.06	204	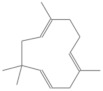
n.i.	28.239	0.08		
β-bisabolene *	28.763	2.32	204	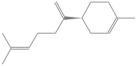
n.i.	28.954	0.06		
δ-cadinene	29.107	0.07	204	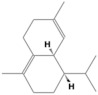
Spathulenol *	30.748	0.15	220	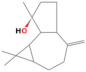
Caryophyllene oxide	30.89	0.24	220	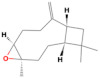
α-cubebene *	31.568	0.02	204	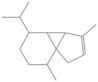
γ-muurolene *	32.043	0.2	204	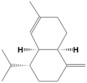
n.i.	32.288	0.05		

n.i.—not identified; *—tentative identification.

**Table 2:** The antioxidant activity of OEO by four different methods.

	ABTS TE μmol/mL	CUPRAC TE μmol/mL	ORAC TE μmol/mL	DPPH Scavenging Activity (%)
OEO	1304 ± 20	5320.1 ± 32	3752.7 ± 28	86 ± 28

**Table 3:** Size and zeta potentials values.

Sample	Particles Size (nm)	PDI	Zeta Potential (mV)
B-PbH	28.4 ± 2.1 (19%) 285.3 ± 11.7 (75%)	1.1059	21.45 ± 0.00
OEO-PbH	20.3 ± 1.9 (64%) 490.2 ± 14.4 (32%)	0.858	−2.52 ± 0.84
OEO	635.1 ± 12.3 (82%)	0.697	0.00 ± 0.00

**Table 4:** Antimicrobial activity of OEO and OEO-PbH.

Bacterial Strains	MIC MBC/MFC (μg/mL)	
	OEO	OEO-PbH
*S. pyogenes* ATCC 19615	5 5	5 5
*S. aureus* ATCC 25923	5 5	10 10
*E. faecalis* ATCC 51299	5 5	10 10
*E. coli* ATCC 25922	40 40	80 80
*P. aeruginosa* ATCC 27853	40 40	80 80
*C. albicans* ATCC 10231	2.5 2.5	2.5 2.5
*C. parapsilosis* ATCC 22019	2.5 2.5	2.5 2.5

**Table 5:** IC_50_ (μg/mL) values of OEO-PbH after stimulation of HaCaT human keratinocytes for different time periods.

IC_50_ (μg/mL)—24 h	IC_50_ (μg/mL)—48 h	IC_50_ (μg/mL)—72 h
93.26	82.65	29.69

## Data Availability

Not applicable.

## Supplementary Materials

The following supporting information can be downloaded at: https://www.mdpi.com/article/10.3390/pharmaceutics14112413/s1, Figure S1: Experimental timeline for the differentiation of human dendritic cells (DCs); Figure S2: Gating strategy (**A**) and FMO controls (**B**) of FACS stainings.
