# Supplementary figures and images for: Phytochemical Characterization and Biological Evaluation of Origanum vulgare L. Essential Oil Formulated as Polymeric Micelles Drug Delivery Systems
Source: Pharmaceutics. 2022 Nov 8;14(11):2413. doi: 10.3390/pharmaceutics14112413 (PMC9693391; doi:10.3390/pharmaceutics14112413)

**A**

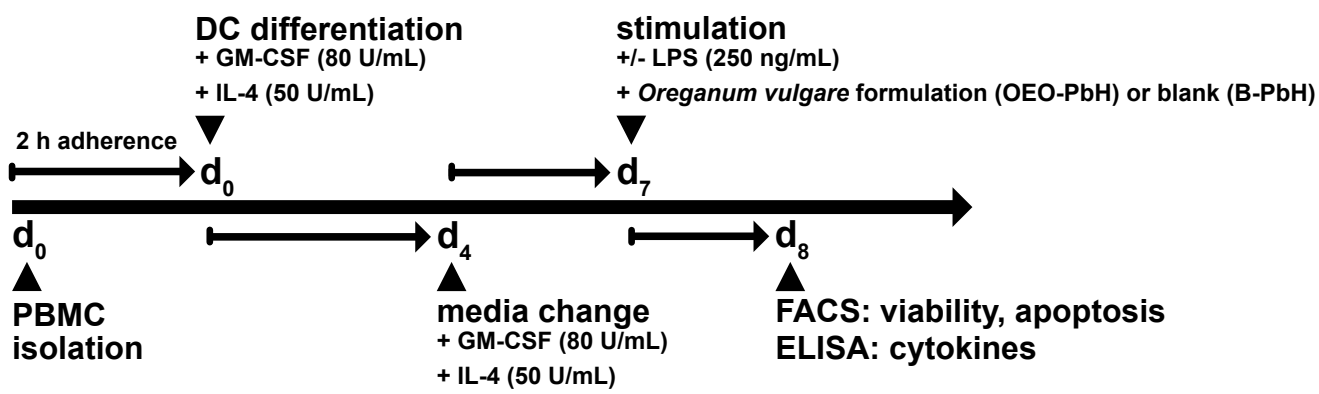

Supplement: Supplementary file 1 [file pharmaceutics-14-02413-s001.zip › Figure S1. timeline.pdf]

**A**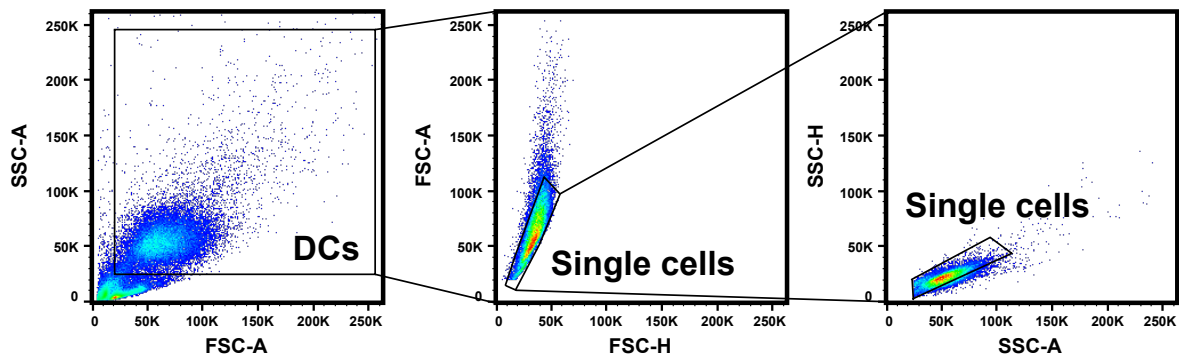**B**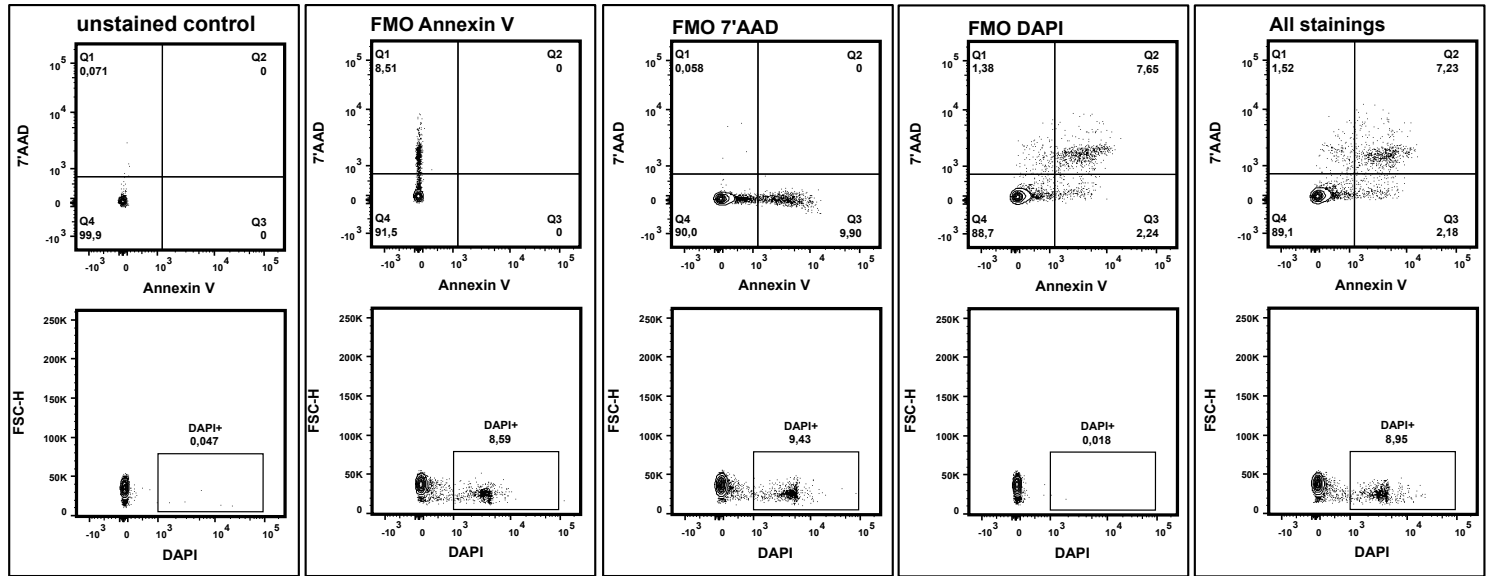

Supplement: Supplementary file 1 [file pharmaceutics-14-02413-s001.zip › Figure S2. Gating_strategy_FMO_stainings.pdf]
